# Supplementary material for: The Prevalence of Delirium in An Older Acute Surgical Population and Its Effect on Outcome
Source: Geriatrics (Basel). 2019 Oct 16;4(4):57. doi: 10.3390/geriatrics4040057 (PMC6960557; doi:10.3390/geriatrics4040057)
Supplement: Supplementary file 1 [file geriatrics-04-00057-s001.pdf]

**The Confusion Assessment Method (CAM) Diagnostic Algorithm**

**Feature 1:** ***Acute Onset and Fluctuating Course***
This feature is usually obtained from a family member or nurse and is shown by positive responses to the following questions: Is there evidence of an acute change in mental status from the patient's baseline? Did the (abnormal) behavior fluctuate during the day, that is, tend to come and go, or increase and decrease in severity?

**Feature 2:** ***Inattention***
This feature is shown by a positive response to the following question: Did the patient have difficulty focusing attention, for example, being easily distractible, or having difficulty keeping track of what was being said?

**Feature 3:** ***Disorganized thinking***
This feature is shown by a positive response to the following question: Was the patient's thinking disorganized or incoherent, such as rambling or irrelevant conversation, unclear or illogical flow of ideas, or unpredictable switching from subject to subject?

**Feature 4:** ***Altered Level of consciousness***
This feature is shown by any answer other than "alert" to the following question:
Overall, how would you rate this patient's level of consciousness? (alert [normal]), vigilant [hyperalert], lethargic [drowsy, easily aroused], stupor [difficult to arouse], or coma [unarousable])

**The diagnosis of delirium by CAM requires the presence of features 1 and 2 and either 3 or 4.**

Figure s1: The Confusion Assessment Method Diagnostic Algorithm.

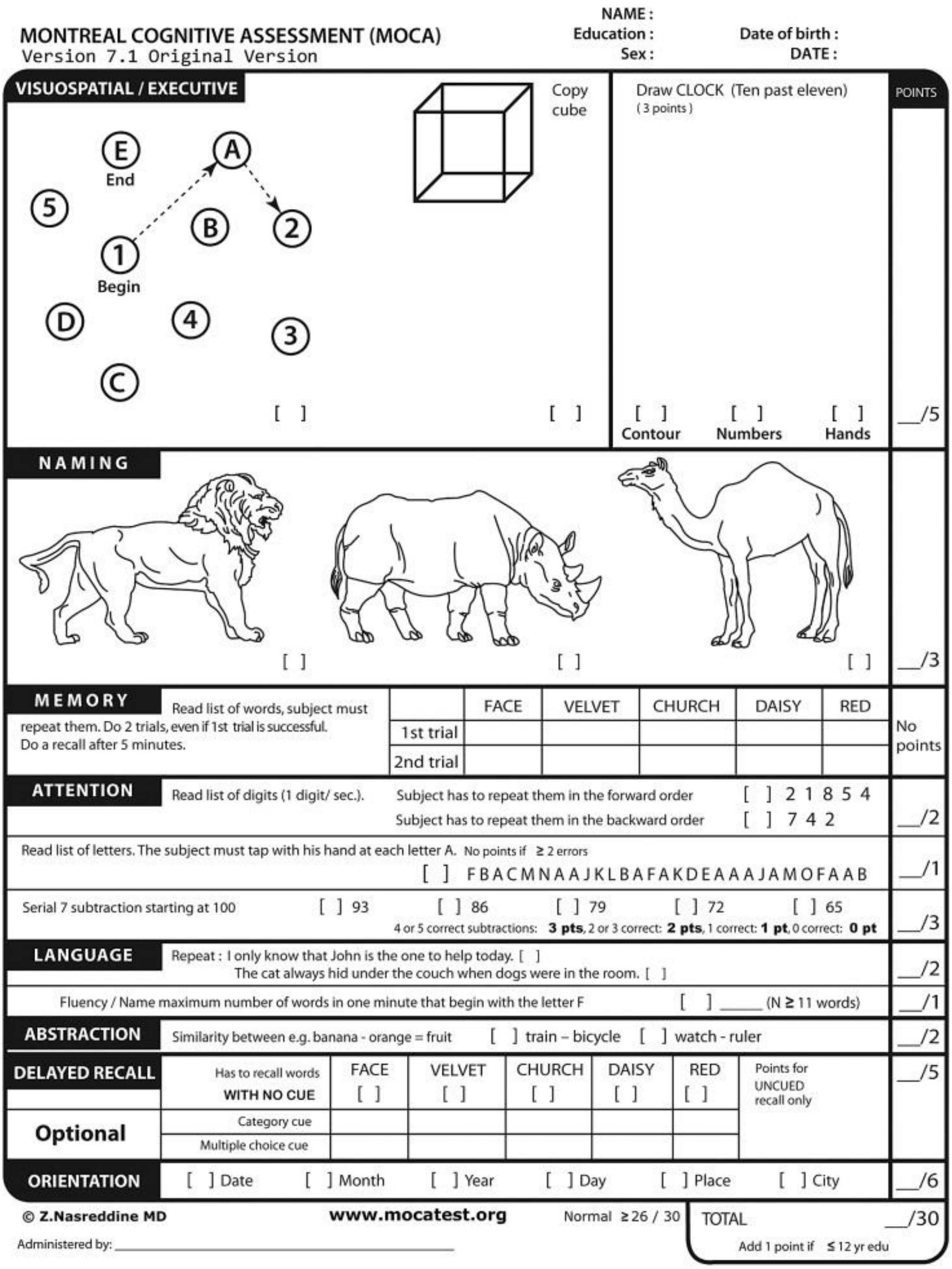

MONTREAL COGNITIVE ASSESSMENT (MOCA)
Version 7.1 Original Version

NAME :
Education :
Sex :
Date of birth :
DATE :

| VISUOSPATIAL / EXECUTIVE | | | POINTS |
|---|---|---|---|
| Trail (5, E End, A, B, 2, 1 Begin, D, 4, 3, C) [ ] | Copy cube [ ] | Draw CLOCK (Ten past eleven) (3 points) [ ] Contour [ ] Numbers [ ] Hands | ___/5 |

| NAMING | | | |
|---|---|---|---|
| [ ] | [ ] | [ ] | ___/3 |

| MEMORY | | FACE | VELVET | CHURCH | DAISY | RED | |
|---|---|---|---|---|---|---|---|
| Read list of words, subject must repeat them. Do 2 trials, even if 1st trial is successful. Do a recall after 5 minutes. | 1st trial | | | | | | No points |
| | 2nd trial | | | | | | |

| ATTENTION | | |
|---|---|---|
| Read list of digits (1 digit/ sec.). | Subject has to repeat them in the forward order [ ] 2 1 8 5 4<br>Subject has to repeat them in the backward order [ ] 7 4 2 | ___/2 |
| Read list of letters. The subject must tap with his hand at each letter A. No points if ≥ 2 errors | [ ] F B A C M N A A J K L B A F A K D E A A A J A M O F A A B | ___/1 |
| Serial 7 subtraction starting at 100 | [ ] 93 [ ] 86 [ ] 79 [ ] 72 [ ] 65<br>4 or 5 correct subtractions: **3 pts**, 2 or 3 correct: **2 pts**, 1 correct: **1 pt**, 0 correct: **0 pt** | ___/3 |

| LANGUAGE | | |
|---|---|---|
| Repeat : | I only know that John is the one to help today. [ ]<br>The cat always hid under the couch when dogs were in the room. [ ] | ___/2 |
| Fluency / Name maximum number of words in one minute that begin with the letter F | [ ] _____ (N ≥ 11 words) | ___/1 |

| ABSTRACTION | | |
|---|---|---|
| Similarity between e.g. banana - orange = fruit | [ ] train – bicycle [ ] watch - ruler | ___/2 |

| DELAYED RECALL | Has to recall words WITH NO CUE | FACE [ ] | VELVET [ ] | CHURCH [ ] | DAISY [ ] | RED [ ] | Points for UNCUED recall only | ___/5 |
|---|---|---|---|---|---|---|---|---|
| Optional | Category cue | | | | | | | |
| | Multiple choice cue | | | | | | | |

| ORIENTATION | | | | | | | |
|---|---|---|---|---|---|---|---|
| | [ ] Date | [ ] Month | [ ] Year | [ ] Day | [ ] Place | [ ] City | ___/6 |

© Z.Nasreddine MD www.mocatest.org Normal ≥ 26 / 30 TOTAL ___/30

Add 1 point if ≤ 12 yr edu

Administered by: ______________________

Figure S2. The Montreal Cognitive Assessment Test (MoCA)

Table S1. Patient characteristics from each of the sites.

| | | Cardiff | North Bristol | Paisley | Central Manchester | Aberdeen | Total |
|---|---|---|---|---|---|---|---|
| | | (n=77) | (n=92) | (n=45) | (n=51) | (n=146) | 411 |
| Sex | Women | 37 | 45 | 28 | 28 | 74 | 212 |
| | Men | 40 | 47 | 17 | 23 | 72 | 199 |
| Age, | mean (SD) | 78.2 (8.5) | 78.6 (9.1) | 76.8 (7.9) | 75.2 (7.6) | 76.7 (7.4) | 77.3 (8.1) |
| Frailty | Not Frail | 51 | 54 | 26 | 45 | 119 | 295 |
| | Frail | 24 | 37 | 19 | 5 | 27 | 112 |
| Operation | No | 72 | 69 | 27 | 39 | 117 | 324 |
| | Yes | 5 | 23 | 11 | 12 | 28 | 79 |
| Haem | Normal | 49 | 44 | 23 | 24 | 88 | 228 |
| | Low | 48 | 48 | 22 | 27 | 58 | 183 |
| Albumin | Normal | 39 | 33 | 17 | 26 | 125 | 240 |
| | Low | 30 | 59 | 28 | 25 | 21 | 171 |
| Diabetes | No | 56 | 77 | 39 | 38 | 120 | 330 |
| | Yes | 19 | 15 | 6 | 13 | 26 | 79 |

Table S2. Length of Hospital stay categorised by the presence of delirium.

| Length of Hospital stay (days) | Not Delirious | Delirious | Total |
|---|---|---|---|
| 1-2 | 78 | 1 | 79 |
| 3-6 | 149 | 14 | 163 |
| 7-13 | 80 | 8 | 88 |
| 14+ | 60 | 12 | 73 |
| Total | 367 | 36 | 403 |
